# Hair androgen concentrations and depressive disorders in adolescents from the general population

**DOI:** 10.1007/s00787-021-01929-w

**Published:** 2022-02-02

**Authors:** Hanna Kische, Catharina Voss, Robin Haring, Theresa Magdalena Ollmann, Lars Pieper, Clemens Kirschbaum, Katja Beesdo-Baum

**Affiliations:** 1 Institute of Clinical Psychology and Psychotherapy, Behavioral Epidemiology, Technische Universität Dresden, Chemnitzer Str. 46, 01187 Dresden, Germany; 2 Faculty of Applied Public Health, European University of Applied Sciences, Rostock, Germany; 3 School of Public Health and Preventive Medicine, Monash University, Melbourne, Australia; 4 Center for Clinical Epidemiology and Longitudinal Studies (CELOS), Institute of Clinical Psychology and Psychotherapy, Technische Universität Dresden, Dresden, Germany; 5 Department of Biopsychology, Technische Universität Dresden, Dresden, Germany

**Keywords:** Hair testosterone, Testosterone, Dehydroepiandrosterone, Depressive symptoms, Major depressive disorder, Epidemiology

## Abstract

Although the link between androgens and depression is well established in adults, the effects of cofactors on this association are less clearly understood, particularly in youth. Epidemiological cohort study of adolescents in Dresden, Germany. Analyses comprised data of 985 individuals assessed at baseline and of 512 individuals at 1-year follow-up. We investigated multivariable regression models for cross-sectional and longitudinal associations of hair testosterone, dehydroepiandrosterone (DHEA), and their cortisol ratios with 12-month diagnoses of major depressive disorder (MDD) and MDD without any anxiety disorder assessed with standardized diagnostic interview (DIA-X-5), and with dimensional depression scores (PHQ-9, PROMIS), separately for males and females. The potential moderating effect of social support was determined. Cross-sectional analyses yielded inverse associations of testosterone and DHEA with MDD and MDD without any anxiety disorders in males. In cross-sectional and longitudinal analyses, baseline ratio cortisol/DHEA was significantly, inversely associated to PROMIS-depression in males. Only cross-sectional associations for ratio cortisol/DHEA and PROMIS-depression remained significant after Bonferroni-Holm correction. No robust associations were observed in female participants. Social support exerted no consistent moderating effect on the investigated association. The present observational cohort study showed no consistent association of hair androgen concentrations with depressive disorders in adolescents. However, findings provide some support for the association between the cortisol/DHEA ratio and depression in males. Longitudinal research designs in large samples are needed to understand the interplay between androgens, depression, and developmental and social factors in youth.

**Supplementary Information:**

The online version contains supplementary material available at 10.1007/s00787-021-01929-w.

## Introduction

During adolescence, the incidence of depressive disorders increases dramatically [12, 16], resulting in overall high 12-month prevalence estimates ranging from 11.3% for major depressive episodes [69], and 7.5% [8] to 12.9% for major depressive disorders [61]. Even higher rates are reported when assessing depression by questionnaire or screening-scales (e.g., Becks Depression Inventory: 49.2% [48]). Impressively, the cumulative incidence of depression rises from 3.8% at 12 years to 36.1% at 17 years in females in a representative sample of the US population [16]. Furthermore, depressive disorders are already linked in adolescence to anxiety disorders, chronic physical conditions as well as mortality [101]. Regarding sex differences, females are more than twice as likely to be affected by depressive disorders compared to males with sex differentiation not occurring before adolescence [83]. Sex differences have been shown to be temporally linked to menarche [74] and to persist throughout reproductive years [107], before attenuating above the age of 65 [3].

The role of distal and proximal risk factors for depressive disorders is increasingly well understood, including evidence that negative life events and chronic psychosocial stress constitute relevant risk factors [7]. Nevertheless, these risk factors do not fully explain the increasing incidence of depressive disorders in adolescence or the emerging gender gap during this crucial developmental period [105].

The link between the role of adolescence and timing of brain development is increasingly understood [97]. In brief, developmental changes in reward circuitry during adolescence increases vulnerability for mental disorders like depression and are in part dependent on sex hormone concentrations, particularly in females [59]. Additionally, the development on hippocampal volumes during adolescence is hypothesized to mediate the association between androgens and the risk for depressive symptoms [30].

Besides the development of brain structures, the response to stressors plays an important role regarding trajectories of psychopathology during adolescence. Sex-specific concentrations of rising sex hormones during adolescence change the stress response as a key factor for the development of depression. Sex hormones modulate the stress response as a part of behavioural and neuronal changes. For estrogens, it is well investigated in adults [2, 50, 72, 80, 92], but there is less research effort for studies with adolescents [73, 107] and evidence stem mainly from animal models [42]. Furthermore and especially androgens contribute to sex differences in the stress response systems and therefore in the development of stress-related disorders like depression [108]. Findings from rodent studies suggest puberty as a critical organizational period, meaning that sex-specific increase in androgen concentrations result in sex-specific androgen-sensitive stress response phenotypes [108]. Further, pre-pubertal gonadectomy increased anxiety-like behaviours in male mice but decreased it in female mice [14]. Even depression-like symptomology increases not only in androgen-deficient rodents, but also in androgen-*receptor*-deficient mice [46].

Regarding epidemiological research on the potential link between androgens and depressive disorders in humans yielded conflicting results [17]. In adults, low androgen concentrations [3, 34, 88], were linked to higher prevalence and incidence of depression in males. In adult women, previous studies also linked high [10, 23, 103], but also low testosterone to depressive disorders [38]. In a previous study, we showed that especially change in testosterone concentrations rather than single-point testosterone levels were associated with depressive symptoms in males [55].

In respect to adolescent epidemiological research, a population-based study with 213 adolescents [41] and a study with 88 children [30] showed that lower testosterone in males was associated with higher levels of anxiety and depressive symptoms. A review by Duke et al. confirmed this link, but simultaneously stressed that this association was not found consistently [25]. Regarding adolescent females, results from the epidemiological Great Smoky Mountains study showed an association of higher testosterone with increased levels of depression in females. Furthermore, Goodyer et al. investigated the ratio of cortisol and DHEA, suggesting that the interplay between the stress hormone cortisol and the potentially protecting DHEA effects on the brain and its interplay with mood [6] could be linked with depression [4, 40]. Although the ratio of cortisol and DHEA was widely examined and linked to depression [6, 49, 63, 68], the theory has been criticized [4] and warrants further investigation before sound conclusions about the interplay between cortisol, DHEA and depression can be drawn [35], especially in adolescents. In addition, increase in DHEA and testosterone during adolescence was linked with increased cortisol concentrations and may affect cortisol regulation during the developmental stage of adolescence [53].

Overall, pubertal timing has been related to increasing psychopathology [67] with early timing contributing to a higher risk for depression [30, 93], at least at an early age [85]. Otherwise, not pubertal timing but advancing puberty itself was associated with higher risk of depression [60]. In addition, it is unclear whether this association is due to changes in hormone levels or to contributing other factors as well. The hormone – depression relationship may be more distinctly present under specific circumstances, like, e.g., with parental history of depression [13], or the absence of social support or in an environment with high social pressure. Wang et al. proposed that the social environment may play an important role in the associations between biological factors like sex hormones and depression [102]. Thus, social support might be a relevant cofactor (moderator) in the association between androgens and depression. It is defined as “having or perceiving to have close others who can provide help or care, particularly during times of stress” [29]. As previously reported, social support can be seen as a coping resource and has a protective role on stress and mental health. It is considered to create a positive individual context independent of stressful environment (principal effect model) or/and to moderate the impact of stress on health (stress-buffering effect) [26, 79]. Social support as family, teacher, and peer support has been shown to reduce depression in maltreated adolescents [33], Chinese adolescents [20], or may be a protective factor against depression [82]. Social support was linked inversely with androgen concentrations in adults with the background idea that evolutionary selection pressure increased the capacity for downregulating testosterone in nurturant contexts as partnering and parenting [37], but studies in adolescents are rare.

In summary, population-based studies of associations between depression and endogenous androgens among both sexes in adolescents are scarce and yielded inconsistent results in part due to heterogeneity of methods and lacking analyses of important cofactors. Thus, the aim of the present study was to investigate cross-sectional and longitudinal associations of hair androgens with depression in a population-based cohort of adolescents, considering in particular comorbid anxiety disorders, pubertal development, and social support. Given that it is not yet clear whether the construct of depressive disorders and the severity of depressive symptoms influence the associations between androgens and depression, we examined both, specific categorical depression diagnoses established via standardized interviews and dimensional depression scores established by self-report symptom scales. Our main hypothesis was that higher androgens in females and lower androgens in males might be associated with depression. The secondary hypothesis was that social support moderates the associations between androgens and depression.

## Methods

### Study population

The Behavior and Mind Health (BeMIND) study is a longitudinal cohort study of a general population sample of adolescents from Dresden, Germany. We recently published details of the study design, procedures, and recruitment [11]. In brief, the BeMIND study aimed to investigate developmental trajectories of mental and behavioural disorders. At this, an age- and sex-stratified random sample of 14–21 years olds was drawn from the population registry of the city of Dresden. Assessments were conducted between 11/2015 and 12/2016 and the smaller-scale 1-year follow-up (FU1) was conducted between 02/2017 and 01/2018. Overall, 1180 adolescents (female: 685) participated in the baseline assessment (response rate: 21.7%; cooperation rate: 43.4%), after written invitation by the study team and informed consent from each participant and from all legal guardians in minors (Details in Beesdo-Baum et al. 2021). Main reasons for non-participation were lack of time and lack of interest. At FU1, N = 776 of the original baseline participants were re-assessed. The mean time between baseline assessment and FU1 was 352.59 (SD 65.8) days. The median time between baseline and FU1 was 337 days, the minimum 261 days, and the maximum 609 days. The study protocol was accepted by the ethics committee of the Technische Universität Dresden (TUD). At baseline, participants were asked to complete a standardized clinical-diagnostic assessment, and an experimental assessment approximately 1 week later in the study centre at TUD, as well as an online questionnaire assessment and an Ecological Momentary Assessment (EMA) between these two personal appointments. Biological and physiological data were collected during the EMA period and at the second personal appointment. At FU1, a similar but less extensive assessment program was completed.

**Fig. 1:**
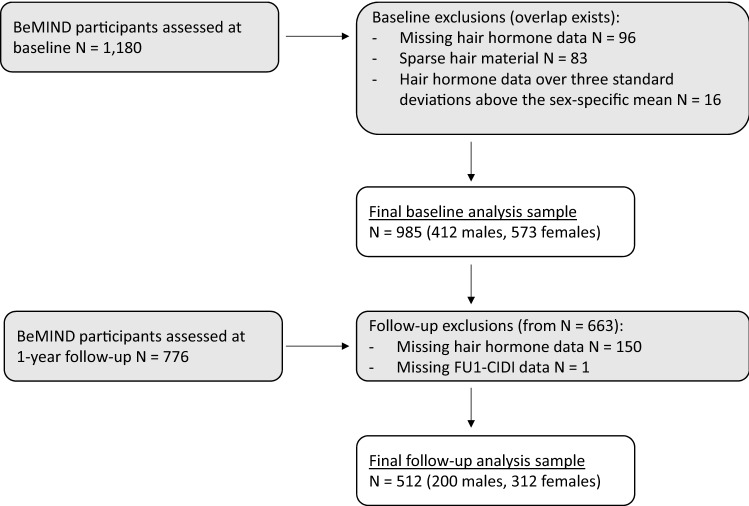
Flow chart of the analyzed study population. Legend: BeMIND, Behavior and Mind Health Study

For the current analysis, we excluded participants with missing testosterone (*N* = 96), missing DHEA (*N* = 1), insufficient hair material (*N* = 83), and with values in testosterone (*N* = 11) and DHEA data (*N* = 5) over three standard deviations above the sex-specific mean. None of the participants used systemic corticoid medication. Finally, valid baseline data were available in 573 females and 412 males (Fig. 1). Regarding age, Tanner Stage, waist circumference, physical activity, current smoking, and alcohol use, no significant differences were found between excluded and included participants for the present analysis.

### Measures

#### Categorial and dimensional assessment of depression

Diagnostic status of every subject was determined at baseline using an updated computer-assisted version of the established standardized Munich Composite International Diagnostic Interview (DIA-X/M-CIDI; [104], providing lifetime and 12-month diagnoses of a range of mental disorders according to DSM-5 [5] criteria (DIA-X-5/D-CIDI; [45]). At FU1, the 12-month interval version of the DIA-X-5 was used, thus FU1 diagnoses refer to occurring disorders during the time between baseline and FU1. For the present analyses we used the following 12-month-diagnoses: Major Depressive Disorder (MDD), and MDD without any comorbid Anxiety Disorder. Any Anxiety Disorder comprised Panic Disorder, Generalized Anxiety Disorder, Social Anxiety Disorder, Agoraphobia, Separation Anxiety Disorder, and Specific Phobia.

Current depression severity was measured using established dimensional scales *Patient Health Questionnaire Depression Module (PHQ-9)* [57]: Prior to the Depression section of the DIA-X-5 at baseline and FU1, participants were asked to rate how they felt in the previous 2 weeks using 9-items reflecting the DSM-IV/DSM-5 criteria for MDD. Each question was scored from 0 (not at all) to 3 (nearly every day), resulting in a possible total score of 0–27.

*Patient Reported Outcomes Measurement Information System for Depression (PROMIS Depression):* The DSM-5 self-rated measure level 2, Depression, Adult item bank for depression [77] in the 8-item short form was applied at the second personal appointment at baseline. The scale does not capture the full range of DSM-defined diagnostic symptoms for MDD. Each question was scored for the past week from 1 (never) to 5 (always), resulting in a range of 8–40 with higher scores indicating a more severe manifestation. PROMIS-Depression was not assessed at FU1 in this form.

#### Androgen and cortisol measures

To obtain markers of long-term testosterone, DHEA, and cortisol secretion, hair samples were taken by trained study personnel at baseline and at FU1. Two hair strands of 3 mm diameter each were cut as close as possible to the scalp from a posterior vertex position. The proximal 3-cm hair segment was used for analyses. Based on an average hair growth rate of 1 cm per month these measurements reflect steroid hormone secretion within 3 months prior to assessment. Hair androgen measurement is seen as a putative measure of systemic, free hormone secretion over a period of months [19, 24]. Analyses were carried out at the laboratory of the Biopsychology Unit at TUD. The preanalytic procedures are described in detail elsewhere [89]. Briefly, samples were washed twice in 2.5-mL isopropanol for 3 min, and steroid hormones were extracted from 7.5 mg of whole, non-pulverized hair using 1.8-mL methanol for 18 h at room temperature. 1.6 mL of the clear supernatant was transferred into a new 2-mL tube. Androgens and cortisol were measured using commercially available chemiluminescence immunoassays with high sensitivity (IBL-International, Hamburg, Germany). The lower limit of detection was 1 pg/mg for cortisol, 0.06 pg/mg for testosterone, and 0.1 pg/mg for DHEA. The intraassay coefficient was < 9%, and the interassay coefficient was < 12%.

#### Moderator variable social support

To provide a comprehensive score for social support we used a composite score including the three questions of the Oslo social support scale (Oslo-3) [17, 66] and the 7-item version of the perceived Social Support Questionnaire (F-SozU) [27], which were both applied at baseline during the online-questionnaire assessment.

Oslo-3: One question was about the number of close confidants, the second about perceived interest and concern from other people and the third question about practical support. These items were added together to yield an additive, composite index from 3 to 14 [56].

F-SozU-7: The 7-item version [28] of the F-SozU [87] assesses general perceived social support with a 5-point Likert scale ranging from 1 (not true at all) to 5 (very true).

Both scores (Oslo-3 and F-SozU-7) were z-standardised, merged, and the composite score was also z-standardised resulting in an overall social support score with higher scores indicating higher levels of perceived social support.

#### Covariates

Sociodemographic information (including information about sex and age) were assessed during the computer-assisted personal interview at the first personal baseline appointment. Physical inactivity, alcohol and smoking habits, and information about hair treatment and medications (antidepressants, oral contraceptive, inhalative corticoid medication) were assessed via personal interview adjacent to hair sampling at the second personal baseline appointment. Education levels refer to the following categories: *low:* attendance at main school (secondary school in Germany with lower secondary education, level 2 according to the ISCED), secondary modern school qualification. *middle:* high-school diploma, attendance of middle school (type of secondary/junior high school for ages 10–16). *high:* academic high school, grammar school in Germany (secondary school ISCED level 3), college, senior technical college, university degree. *other:* all other type of school. Alcohol consumption was categorized in five self-reported categories (never, on special occasions, once or twice in month, once or twice in week, daily/almost daily). Self-reported information about smoking habits were categorized in current smoker and no current smoker including never smoker. The use of oral contraceptive was assessed based on a questionnaire. Hair and hair cosmetic-related information (hair color, frequency of hair cleaning, and hair treatment with heat) was obtained via the following questions: “What is your natural hair color? How often do you wash your hair per week? Do you have a perm, or do you use a hair straightener or a hairdryer? If yes, when?”. Waist circumference was measured utilizing a tape midway between the lower rib margin and the iliac crest in the horizontal plane rounded to the nearest mm. Questions about menstruation status in detail were only available at FU1. At this, menstrual status was assessed by self-reported information about date and length of last menstrual cycle and regularity of cycles. Pubertal maturation was assessed via sex-specific questions in the online questionnaire. At this, individuals were told to rate themselves on sex-appropriate schematic drawings (females: breasts and pubic hair development; males: pubic hair and genitalia development). These self-ratings were averaged to yield “Tanner stages”, meaning individual characteristic from prepubertal to adult (1–5) [90].

### Statistical analysis

Categorical data are given as weighted percentage and continuous data as mean (standard error) or median (p25th, p75th). We applied sample weights in all statistical models to equal sex and age distribution between the sample and target population of the 14–21-year-old individuals living in Dresden [11]. Given a number of literature indications, significant differences in hormone levels between males and females in our sample, and a number of significant interaction terms between hormones and sex, all analyses were performed separately in males and females.

First, we conducted cross-sectional and longitudinal analyses with logistic regression models for dichotomous depression outcome variables and linear regression models for dimensional depression outcome variables using hormones as predictors. Effects in logistic regression models were reported as odds ratios (OR) with their 95% confidence interval (CI) and in linear regression models as *b*-coefficients and their 95% CI. We checked the assumptions for linear regression models, by checking (1) the linear association of predictor and outcome, (2) outliners, (3) the independence of residuals, (4) for multicollinearity of covariates, and (5) homoscedasticity. Additionally, we used QQ plots to test the normality of regression residuals. Further, we checked the assumptions for logistic regression models and refer to the limitation section for the assumption of minimal number of cases per predictor.

In longitudinal analyses, we presented as predictors (a) baseline hormone concentrations and (b) change in hormone concentrations from baseline to FU1. Hormone change was defined as absolute difference between baseline and 1-year FU hormone concentration. Multivariable regression models were adjusted for age or Tanner Stage, respectively, waist circumference, smoking status, alcohol consumption, physical inactivity, hair color, frequency of hair cleaning, and hair treatment with heat, and oral contraceptives (only in analyses with females). We additionally included analyses with standardized betas (using standardized dependent and independent variables in linear regressions) and standardized ORs (using standardized independent variables in logistic regressions). To investigate potential moderating effects of social support on the investigated associations, we used regression models with interaction between androgen variables and a z-standardized composite score for social support (Oslo-3 and F-SozU-7) to estimate additive interaction effects via margins. All interaction terms with standardized variables have been computed with relevant variables, which were standardized before the analyses.

Several separate sensitivity analyses were performed. First, we added four specific confounders to the multivariable models, respectively: possible pregnancy, menstruation status, use of inhalated corticoid medication and use of antidepressants. These confounders have been found to have an impact on the outcomes and predictors (= confounder), but either with no high evidence (for one of them) and not with such high effects compared to the other confounders initially used in the main multivariable analyses or the variables itself had limitations (Supplement 2). Therefore, there were only included in the following sensitivity analyses: Multivariable regression analyses among women were adjusted for possible pregnancy (*N* = 9) and in longitudinal analyses with change in hormone concentrations for menstruation status (as this information were not available at baseline). Furthermore, analyses in the full sample were adjusted for inhalated corticoid medication and use of antidepressants.

Second, besides the main analyses focussing linear effects, we wanted to test possible curvilinear associations. Analyses with hair androgen concentrations were categorized in tertiles with tertile 2 as reference to detect possible non-linear associations as an additional insight regarding our hypothesis that very low or very high concentrations may be associated with depression in females, and therefore used tertile 2 as reference tertile. A *p*-value < 0.05 was considered statistically significant. We performed additionally Bonferroni-Holm Corrections for the main effects. All statistical analyses were performed with Stata 14.2 (StataCorp. 2015. *Stata Statistical Software: Release 14*. College Station, TX: StataCorp LP).

## Results

### Sample characteristics

Weighted, baseline characteristics of the analyzed study sample are presented in Table 1, separately for males and females. Females showed lower hair testosterone and hair DHEA concentrations, but higher scores in PHQ-9, PROMIS Depression and perceived social support compared to males. *N* = 56 females (11.5% of all females) used oral contraceptives. Concerning baseline 12-month depression diagnoses in the present analysis sample the following numbers of individuals were diagnosed with MDD (males *N* = 11, females *N* = 30), and MDD without any comorbid anxiety disorders (males *N* = 6, females *N* = 14).

**Table 1:** Baseline characteristics of the study population

Variable	Total Sample (*N* = 985)	Males (*N* = 412)	Females (*N* = 573)	*p*-value*
Age, years	17.9 (0.07)	18.0 (0.11)	17.9 (0.10)	0.425
Sex, female, %	48.1	0	100	
Education, %				0.190
Low	2.1	3.2	1.0	
Middle	16.8	15.8	17.7	
High	78.1	78.1	78.2	
Other	2.9	2.7	3.1	
12-Month MDD, %	4.3	3.1	5.6	0.042
12-Month MDD without any anxiety disorder, %	2.2	1.6	2.8	0.257
PHQ-9 Depression Score, mean (SD)	4.19 (0.12)	3.71 (0.17)	4.71 (0.18)	< 0.001
PROMIS Depression Score, mean (SD)	11.04 (0.16)	10.28 (0.21)	11.86 (0.23)	< 0.001
Hair testosterone, ng/mg	0.76 (0.02)	0.96 (0.04)	0.54 (0.02)	< 0.001
Change in hair testosterone, ng/mg	– 0.02 (0.04)	– 0.06 (0.06)	0.01 (0.04)	0.791
Hair DHEA, ng/mg	36.5 (1.84)	42.52 (3.29)	29.9 (1.28)	< 0.001
Change in hair DHEA, ng/mg	– 6.7 (2.47)	– 10.55 (4.58)	– 3.86 (1.8)	0.933
Hair cortisol, ng/mg	7.26 (0.47)	6.78 (0.83)	7.78 (0.39)	< 0.001
Ratio Cortisol/Testosterone	27.7 (2.92)	21.64 (5.87)	40.0 (5.28)	< 0.001
Ratio Cortisol/DHEA	0.35 (0.02)	0.29 (0.02)	0.42 (0.03)	< 0.001
Waist circumference, cm	78.1 (0.39)	80.8 (0.61)	75.1 (0.46)	< 0.001
Current smoker, %	20.6	22.4	18.6	0.253
Physically inactive, %	36.2	34.5	38.1	0.112
No alcohol use, %	20.1	18.9	21.3	< 0.001
Use of oral contraceptives, %	5.5	0	11.5	< 0.001
Oslo-3-Score, mean (SD)	10.9 (0.06)	10.7 (0.11)	11.2 (0.09)	< 0.001
F-SozU-7 Score, mean (SD)	4.19 (0.02)	4.04 (0.04)	4.35 (0.03)	< 0.001
Social Support standardized composite score, mean (SD)	– 0.012 (0.036)	– 0.227 (0.060)	0.210 (0.037)	< 0.001
Tanner Stage, %				0.056
1	–	–	–	
2	0.4	0.7	0.1	
3	4.8	5.5	4.1	
4	19.1	19.5	18.6	
5	75.6	74.1	77.1	

Data are weighted percentages, or weighted means (SE). Any depressive disorder “ includes Major Depressive Disorder and Persistent Depressive Disorder (Dysthymia). *Statistical comparisons were performed with χ 2 test (nominal data) or Mann–Whitney-*U*-test (continuous data). No alcohol use: participants with response “never” on the question how often they drank alcohol in the last three months. The social support score is a z-standardized composite score including z-standardized Oslo-3 and z-standardized F-SozU-7

*DHEA* Dehydroepiandrosterone, *MDD* major depressive disorder, *GAD* generalized anxiety disorder, *PHQ* patient health questionnaire, *PROMIS* patient reported outcomes measurement information system, *F-SozU* perceived social support questionnaire

FU1 12-month diagnoses comprise *N* = 21 for MDD, and *N* = 13 for MDD without any comorbid anxiety disorders (supplementary table 1). Regarding the key variables (androgens, depressive disorders, age, sex, social support) and other characteristic variables (social class, any anxiety disorder, any mental health care use, living arrangement, any chronic disease) no significant difference was found between adolescents participating in the FU and drop-out from baseline to FU1. The only detectable difference in drop-out analyses for this manuscript was found for educational status (low: Odds Ratio (OR) = 3.0, 95% confidence interval (CI) = 1.2 – 7.1, *p* = 0.001), middle: OR = 1.7, 95% CI = 1.3 – 2.3, *p* = 0.012), high (Ref), other: OR = 2.2, 95% CI = 1.0 – 3.7, *p* = 0.027).

Regarding separate analyses in males and females, hair testosterone and hair DHEA in pg/mg differ significantly (*p* < 0.01) in males (higher testosterone and DHEA levels) compared to females (lower testosterone and DHEA levels). In regard of change of hair testosterone, a slight decrease over the 1-year FU was observed in males but not in females. Hair DHEA decreases over the 1-year FU in both sexes. Based on a number of significant interaction terms regarding sex in the full sample (supplementary table 2) all analyses were conducted separately in males and females.

### Cross-sectional associations of hair androgen measures with depression at baseline

Table 2 presents cross-sectional associations of baseline androgens and hormone ratios with depression. We found significant inverse associations of testosterone and DHEA with MDD in males (age-adjusted, testosterone: OR: 0.23; 95% CI 0.03–0.98*. DHEA*: OR: 0.96; 95% CI 0.91–0.99), but not in females. In males, the ratio cortisol/testosterone was inversely associated with PHQ-9 depression score. None of the reported cross-sectional associations were observed in females. After Bonferroni-Holm Correction only the association of PROMIS with ratio cortisol/DHEA in males remained statistically significant (age-adjusted, b-coefficient, 95 CI  – 0.01 ( – 0.02;  – 0.004)*, and multivariable (mv)-adjusted, b-coefficient, 95% CI  – 0.01 ( – 0.01;  – 0.007)*). This association was also statistically significant in standardized analyses (after Bonferroni-Holm correction: age-adjusted, ß-coefficient, 95 CI  – 0.10 ( – 0.16;  – 0.03)*, and multivariable (mv)-adjusted, ß-coefficient, 95% CI  – 0.11 ( – 0.19;  – 0.03)*). Effects of standardized results are shown in supplementary Table 3.

**Table 2:** Cross-sectional associations of baseline hair steroid hormones with baseline major depression and depressive symptoms in males and females

Baseline	Testosterone		DHEA		Cortisol/Testosterone		Cortisol/DHEA	
	Males	Females	Males	Females	Males	Females	Males	Females
MDD	Odds Ratio (95% CI)							
Age-adjusted	0.23 (0.03; 0.98)*	1.46 (0.73; 2.94)	0.96 (0.91; 0.99)*	0.99 (0.98; 1.00)	0.99 (0.99; 1.02)	0.99 (0.99; 1.01)	1.08 (0.62; 1.88)	0.94 (0.65; 1.35)
Multivariable-adjusted	0.31 (0.05; 1.60)	1.12 (0.49; 2.56)	0.95 (0.89; 1.01)	0.99 (0.97; 1.00)	0.99 (0.99; 1.02)	0.99 (0.99; 1.00)	1.49 (0.88; 2.53)	0.96 (0.63; 1.45)
MDD without any anxiety disorder								
Age-adjusted	0.19 (0.01; 0.86)*	1.57 (0.83; 2.93)	0.97 (0.90; 1.04)	1.00 (0.99; 1.00)	0.99 (0.99; 1.01)	0.99 (0.99; 1.00)	1.34 (0.85; 2.13)	0.86 (0.42; 1.76)
Multivariable-adjusted	0.20 (0.02; 1.67)	1.69 (0.83; 3.43)	0.97 (0.90; 1.05)	1.00 (0.99; 1.01)	0.99 (0.99; 1.02)	0.99 (0.99; 1.00)	1.35 (0.69; 2.66)	0.96 (0.54; 1.70)
	b-coefficients (95% CI)							
PHQ-9 depression score								
Age-adjusted	0.02 (0.005; 0.04)*	0.01 ( – 0.007; 0.02)	0.53 ( – 1.73; 2.80)	0.21 ( – 0.32; 0.74)	-2.29 ( – 4.51; – 0.08)*	– 29.4 ( – 71.5; 12.6)	– 0.009 (-0.01; 0.004)	– 0.006 (-0.01; 0.004)
Multivariable-adjusted	0.01 ( – 0.01; 0.03)	0.003 ( – 0.008; 0.01)	– 0.44 ( – 2.81; 1.91)	0.003 ( – 0.59; 0.60)	– 2.02 ( – 3.84; – 0.21)*	– 42.7 ( – 92.9; 22.5)	– 0.008 ( – 0.02; 0.006)	– 0.003 ( – 0.01; 0.006)
PROMIS depression								
Age-adjusted	0.01 ( – 0.01; 0.02)	0.001 ( – 0.005; 0.008)	0.36 ( – 1.66; 39)	– 0.06 ( – 0.45; 0.31)	– 1.61 ( – 3.36; 0.13)	– 5.60 ( – 15.1; 3.85)	– 0.01 ( – 0.02; – 0.004)**	– 0.01 ( – 0.01; 0.007)
Multivariable-adjusted	0.006 ( – 0.01; 0.03)	0.001 ( – 0.006; 0.007)	0.26 ( – 2.23; 2.77)	– 0.24 ( – 0.65; 0.17)	– 1.75 ( – 3.90; 0.41)	– 6.65 ( – 21.3; 8.00)	– 0.01 ( – 0.01; – 0.007)**	– 0.001 ( – 0.01; 0.009)

Data are weighted unstandardized odds ratios (from logistic regressions) or b-coefficients (from linear regressions) and their 95% confidence interval, with *p* < 0.05 marked as *, *p* < 0.01 marked as **, and p < 0.001 marked as ***. For standardized results, see supplementary Table 4a. After Bonferroni-Holm Correction only the bold marked results remain statistically significant with PROMIS – ratio cortisol/DHEA in males, age-adjusted:  – 0.01 ( – 0.02;  – 0.004)*, and multivariable-adjusted:  – 0.01 ( – 0.01;  – 0.007)*. The multivariable model was adjusted for Tanner Stage, waist circumference, smoking status, physical inactivity, alcohol consumption, hair color, frequency of hair cleaning, and hair treatment with heat, and oral contraceptives (females). MDD, males *N* = 11, comparison group *N* = 401. MDD, females *N* = 30, comparison group *N* = 543. MDD, without any anxiety disorder males *N* = 6, comparison group *N* = 406. MDD, without any anxiety disorder females *N* = 14, comparison group *N* = 559. PHQ-9, males *N* = 412, females *N* = 571. PROMIS-Depression, males N = 401, females *N* = 565.DHEA, Dehydroepiandrosterone. Effect sizes (Cohen`s d) in males are for testosterone and MDD/MDD without any anxiety disorders: 0.47/0.50, for DHEA: 0.39/0.37, for cortisol/testosterone: 0.21/0.25, for cortisol/DHEA and MDD: 0.23/0.27. Effect sizes (Cohen`s d) in females are for testosterone and MDD/MDD without any anxiety disorders: 0.17/0.21, for DHEA: 0.16/0.08, for cortisol/testosterone: 0.26/0.25, for cortisol/DHEA and MDD: 0.26/0.29. MDD, Major Depressive Disorder. PHQ, Patient Health Questionnaire. PROMIS, Patient Reported Outcomes Measurement Information System. CI, confidence interval

### Longitudinal associations of hair androgens at baseline and change in hair androgens with depression at follow-up

No significant longitudinal associations of baseline testosterone or DHEA with FU1 depression were observed in multivariable models (Table 3). Analyses of baseline ratios, however, revealed a significant association of cortisol/testosterone (mv-adjusted OR: 0.96; 95% CI 0.92–0.99) and cortisol/DHEA with FU1 MDD (mv-adjusted OR: 0.22; 95% CI 0.04–0.83) in females, but not in males. Similarly, to cross-sectional analyses, baseline cortisol/testosterone (mv-adjusted, b-coefficient: -0.002; 95% CI  – 0.004 to  – 0.001), but additionally cortisol/DHEA (mv-adjusted, b-coefficient:  – 0.54; 95% CI  – 0.90 to  – 0.17) and ratio changes were significantly associated to PHQ-9 depression score at FU1 in males, but not in females. After Bonferroni-Holm Correction none of the results remained statistically significant. Effects of standardized results are shown in supplementary Table 4.

**Table 3:** Longitudinal associations of baseline hair steroid hormones and change in hair steroid hormones with follow-up major depression and depressive symptoms and males and females

	Testosterone		DHEA		Cortisol/Testosterone		Cortisol/DHEA	
	Males	Females	Males	Females	Males	Females	Males	Females
	Odds ratio (95% CI)							
FU-MDD								
Baseline hormone conc	1.03 (0.19; 5.37)	1.11 (0.45; 2.73)	0.91 (0.81; 1.02)	0.99 (0.97; 1.01)	0.95 (0.86; 1.06)	0.96 (0.92; 0.99)*	0.72 (0.38; 1.34)	0.22 (0.04; 0.83)*
Hormone change	0.84 (0.31; 2.33)^a^	0.74 (0.37; 1.47)	0.99 (0.98; 1.02)	0.99 (0.98; 1.01)	1.00 (0.99; 1.01)	0.99 (0.98; 1.01)	0.92 (0.37; 2.31)	0.94 (0.88; 1.02)
FU-MDD without any anxiety disorder								
Baseline hormone conc	0.64 (0.04; 9.53)	0.68 (0.20; 2.30)	0.93 (0.84; 1.03)	0.98 (0.96; 1.01)	0.96 (0.89; 1.03)	0.97 (0.94; 1.00)	0.21 (0.01; 3.62)	0.18 (0.02; 1.44)
Hormone change	0.73 (0.39; 1.38)	0.76 (0.34; 1.32)	0.94 (0.81; 1.03)	0.99 (0.98; 1.01)	1.00 (0.99; 1.01)	1.01 (0.99; 1.01)	1.02 (0.97; 1.07)	0.86 (0.63; 1.17)
	b-coefficient (95% CI)							
FU-PHQ-9 Depression								
Baseline hormone conc	0.14 ( – 0.32; 0.61)	– 0.03 ( – 0.83; 0.76)	– 0.002 ( – 0.007; 0.003)	0.002 ( – 0.008; 0.008)	– 0.002 ( – 0.004; – 0.001)*	0.0003 ( – 0.001;0.001)	– 0.54 ( – 0.90; – 0.17)*	– 0.22 ( – 0.04; 0.19)
Hormone change	– 0.19 ( – 0.58; 0.19)	– 0.25 ( – 0.5; 0.08)	0.001 ( – 0.002; 0.005)	– 0.003 ( – 0.01; 0.004)	– 0.001 ( – 0.001; – 0.001)*	0.004 ( – 0.001; 0.008)	– 0.05 ( – 0.07; – 0.02)*	– 0.05 ( – 0.11; 0.001)

Data are weighted, unstandardized odds ratios (from logistic regressions) or b-coefficients (from linear regressions), respectively, and their 95% confidence interval, with *p* < 0.05 marked as *, *p* < 0.01 marked as **, and *p* < 0.001 marked as ***.^a^significant in age-adjusted models and multivariable models without Tanner Stage. For standardized results, see supplementary Table 5a. After Bonferroni-Holm Correction none of the results remained statistically significant. Models were adjusted for Tanner Stage, waist circumference, smoking status, physical inactivity, alcohol consumption, hair color, frequency of hair cleaning, and hair treatment with heat, and oral contraceptives (females). Hormone change is the change of hormone concentration from baseline to 1-year follow-up. PROMIS-Depression was not available at FU1. Effect sizes (Cohen`s d) in males are for testosterone and MDD/MDD without any anxiety disorders: 0.46/0.41, for DHEA: 0.13/0.11, for cortisol/testosterone: 0.19/0.19, for cortisol/DHEA and MDD: 0.21/0.21. Effect sizes (Cohen`s d) in females are for testosterone and MDD/MDD without any anxiety disorders: 0.15/0.18, for DHEA: 0.02/0.07, for cortisol/testosterone: 0.07/0.15, for cortisol/DHEA and MDD: 0.06/0.05

DHEA, Dehydroepiandrosterone. MDD, Major Depressive Disorder. GAD, Generalized Anxiety Disorder. PHQ, Patient Health Questionnaire. PROMIS, Patient Reported Outcomes Measurement Information System. CI, confidence interval, FU, follow-up

### Sensitivity analyses

In *categorized* analyses using hair androgen tertiles, mv-adjusted models revealed significant inverse association of MDD (Tertile 1 vs. Tertile 2 (Ref.), OR: 0.34; 95% CI 0.12–0.98), MDD without any anxiety disorder, and PHQ-9 depression score with DHEA in females (supplementary Table 5). After Bonferroni-Holm Correction, none of the results remained statistically significant.

The performed sensitivity analyses (*pregnancy, menstruation status, inhalative corticoid medication, antidepressants*) did not change the revealed estimates substantially.

### Moderator analyses

When analyzing the interaction androgen social support no consistent significant moderating effect was observed. The only significant interaction effect emerged in the association between DHEA and MDD in males (main effect DHEA with MDD: OR: 0.96; 95% CI 0.92–1.00; Interaction testosterone social support: OR: 1.02; 95% CI 1.01–1.04) but was rendered non-significant after Bonferroni-Holm Correction. The results are shown in supplementary Table 3 and are represented as an additive interaction, meaning the change of risk difference between androgens and depression if social support scale increases by 1 (supplementary Table 6).

## Discussion

The present population-based study examined the association between hair testosterone, DHEA and its cortisol ratios with depression in adolescents. There was slight evidence for an association between testosterone and MDD in males, however, the only robust association was found between the cortisol/DHEA ratio and depressive symptoms in males. After Bonferroni-Holm correction, none of the investigated associations were present in females. To our knowledge, this is the first epidemiological study with adolescents analysing the potential moderating effect of social support in this association. However, contrary to our expectations, we did not observe a consistent moderation effect of social support.

### Females

The present null findings among females are in line with most previous research reporting no association between androgens and depression in female adolescents [41, 60]. Certainly, it was suggested that the increase in depression prevalence during adolescence in females is in part explained by sex hormones [81]. Accordingly, some authors reported higher testosterone concentrations as a predictor for depression in females [22] or lower salivary testosterone concentrations in female adolescent patients. In general, it is assumed that effects in patients [58, 64, 65, 103] may be far less difficult to detect compared with participants from the general population [44] due to more severe and persistent symptoms and higher psychological strain of MDD implying increased pathophysiological processes. A recent meta-analysis and mendelian randomization study suggested that there is indeed an association between testosterone and depression. However, the study could not find a causal relationship and suggested that the direction of the association may be due to menopausal status [62]. Considering that this study included adult women and stressed that the association between testosterone and depression is dependent on menopausal status, the results cannot be directly transferred to the present study. Taken together, both studies highlight the importance of investigating the menopausal status, stages of puberty, and menstrual cycle status in future studies when examining androgens in depressive disorders.

Differences in the results regarding the association between androgens and depression may be additionally due to methodological issues like different laboratory methods (immunoassay versus liquid chromatography-tandem mass spectrometry). Most previous studies measured androgens in saliva or blood for a short-term measurement with diurnal fluctuations. In the present study, a cumulative long-term measurement for hair androgens was assessed, providing more stable outcomes compared with single-point short-term measurement [19, 70].

Furthermore, previous findings hypothesized, that for young females, not the inter-individual differences in androgen concentrations, but the intra-individual change in androgen concentrations also in saliva samples may be an important factor for pathological development of mental disorders [41]. Thus, we performed longitudinal analyses of intraindividual androgen change from baseline to follow-up but did not observe consistent associations. Certainly, it might be that instead of androgens, endogenous estrogens, the conversion of DHEA in estrogens or exogenous estrogens via contraceptives [86] play a more important role in females regarding development and progression of depression, although some authors underline the impact of testosterone only [22].

### Males

The link between androgens and depression was previously observed especially in older men [34, 55], but also in adolescent males [41]. In line with other reports [25, 60], we could not find consistent and strong associations between androgens and depression. Nevertheless, we observed weak inverse associations between androgens and MDD in the present sample but only before corrections for multiple testing and with small effect sizes. Biological mechanisms such as androgen receptors in amygdala, hippocampus, and neocortex, androgens modulation activity and interconnectivity of prefrontal cortex and amygdala in depressive disorders [99], and the influence of pathological imbalance of androgens and cortisol on HPA-axis may in part explain the role of androgens in development and course of depression.

When using diagnosis compared with dimensional scores, the observed effects were more pronounced. This might be due to a more pervasive symptom pattern in CIDI-diagnosed participants compared with just a dimensional rating of symptoms.

Our analyses with the ratio cortisol/androgen yielded no significant results, except evidence for an inverse association between cortisol/androgen and depressive symptoms in male adolescents, which remained statistically significant after correction for multiple testing in cross-sectional analyses. In previous studies, state elevated cortisol/DHEA was related to depression in patients [39, 40, 106]. We cannot fully explain the different directions of this effect, but this might be due to the different measurement points of hormones. Goodyer et al. referred to morning hormone levels with elevated cortisol/DHEA ratio (meaning elevated cortisol and/or decreased DHEA). This is understandable as an elevated cortisol awakening response was associated with a higher risk of developing MDD [1] and patients with MDD showed higher CAR than matched controls [91] in other studies examining cortisol and depression. In contrast to these studies, the present hormone ratios stem from hair samples providing long-term measures. At this, there is evidence for decreased accumulated cortisol levels in chronic depression [36], which would result in a decreased cortisol/DHEA ratio and thus an inverse association. Further, although PHQ-9 and PROMIS-Depression capture depressive symptoms and demonstrated strong convergent validity [52, 78], our results of PHQ-9 and PROMIS-Depression with steroid hormones had the same direction but differ in its significancy. This might be due to small constructive differences of the questionnaires (survey period (PHQ – 2 weeks, PROMIS -7 days, different item formulation) in connection with small effect sizes and small statistical power. However, the present results add to the inconclusive picture in previous literature as cortisol/androgen was related to depressive symptoms, but not to MDD diagnosis. Indeed, DHEA was identified as an antagonist of cortisol with antidepressant effects, but only in patient-based studies or samples with older adults [51]. Thus, it is not yet clear how and under which conditions DHEA or its ratio with cortisol contribute to depression.

The small, but significant effect in the present study underlines the importance to investigate additional subgroups [4] being especially vulnerable to changes in hormone milieus linked to incident depression. Such vulnerable subgroup could be determined in imminent research and might include adolescents with an early or particularly late puberty onset, with parental vulnerability for depressive and anxiety disorders, or with high social-emotional stress burden.

### Social support

Recent studies suggest that social costs in social situations is linked with endocrine dysfunction including an imbalanced DHEA and cortisol response, and an inverse association between social withdrawal and testosterone concentrations in adolescent males was observed by Hayashi et al. [43]. Given additional evidence for an impact of social pressure on the association between pubertal development and depression [102], and the general evidence that low social support is associated with depression, we assumed a moderating effect of the cofactor social support but could not find a consistent effect. There is no consistent, validated score for social pressure in previous literature and concepts of social support are mixed. We used a composite score for social support, including the Oslo-3 score [56] and the F-SozU-7 score [27] to cover social support comprehensively. However, the present findings do not provide supporting evidence for a significant moderating role of social support in the association between androgens and depression in adolescents. The assessed composite score for social support in the present study might not be specific enough to determine effects in this specific relationship. Particularly social pressure may not be sufficiently covered by social support constructs. Thus, questions about social acceptance in school, gender role and mobbing due to body characteristics during adolescence, might be expedient and should be developed as an aggregate score for future research questions.

### Time of adolescence

There exist several theories, why adolescence is such a vulnerable phase to develop depression, especially in females, including psychosocial theories, higher stress exposure on a maturing HPA axis, sex hormones interacting with the stress system and neurotransmitter system implicating protective effect of androgens [71]. These effects together with a predisposition or psychosocial trigger may increase the risk for adolescent depression onset. In the present study, we observed a slight decrease in the effect when adjusting for Tanner Stage. However, with the limitation of a study population late in pubertal development with no participants in Tanner Stage one and the small change in effects, we do not interpret these findings as an important influence of puberty. Considering the chronological development of depression, the time of adolescence is linked with internalizing disorders, thus, it would be worth focussing on the complex interplay with puberty in future research.

### Strengths and limitations

Strengths of the present study include the population-based approach and the longitudinal design with a 1-year follow-up, using an appropriate time interval for the detection of biomarker effects [100]. In addition to dimensional depression scales, we used categorical diagnoses assessed with DSM-5-based computer-assisted personal standardised interview.

Limitations might have arisen from the regional BeMIND sample (Dresden, Germany) (high educational level), which restricts the generalization of the results to adolescents with other educational or social backgrounds. Separate outcome analysis in males and females yielded small subgroup samples with limited statistical power to detect significant associations, but comparable in size with previous studies [1]. More cases with specific diagnoses can be assumed in future analyses when the 3-year FU data in BeMIND become available.

Actually, the hair hormone measurement is a strength of this study (Assessment of sex hormones via hair samples, providing a long-term measurement. Using the simple and non-invasive collection of hair samples by trained and supervised study personnel ensures a standardized sampling method in contrast to salivary samples with diurnal fluctuation). Nevertheless, we found an unexpected decrease in DHEA levels over the 1-year follow-up. It is a common challenge when analyzing hair androgens due to the very low androgen levels in females which can be found frequently near to the lower limit of detection. Nevertheless, the validity of hair hormone measurement in the present study is confirmed by the significant differences in hair hormone levels in females compared to males and it is a validated routine method in the laboratory of the Biopsychology unit at TUD [89]. In addition, high quality studies with hair hormone measurement are currently evolving with associations between hair hormones and mental disorders (exemplarily: [15, 54, 76, 94, 95] or considering methodological issues [9, 31, 47]. Regarding the methodological challenges, diurnal steroid hormone levels (e.g., measured via saliva or urine samples) and longer-term steroid hormone levels (measured via hair samples) should be investigated congruently in longitudinal studies to compare the characteristics of the sampling methods [18].

An alternative explanation for the weak associations in the present study relates to the genetic background, particularly the androgen receptor CAG repeat. There is evidence that the association between androgens and depression are significant only when the CAG repeat length can be considered [21, 84], also in adolescents [96]. The Massachusetts Male Aging Study suggested that CAG repeat length as a genetic marker was associated with androgen receptor activity and low versus high testosterone was associated with an increased likelihood of depressive symptoms in men with shorter CAG repeat length [84]. Since CAG repeat data were not assessed in the BeMIND-Study, we were not able to examine the mediating role of the genetics.

The findings in this study have to be interpreted in light of the multiple testing problem and related type I error. The main analyses were conducted without correction for multiple testing, as *p* value adjustment may reduce the chance of type I errors, but in parallel, it increases the chance of type II errors [32]. Regarding the large number of conducted tests (which are typical for epidemiological research), we presented a Bonferroni-Holm-Correction. The significant results regarding androgens and dichotomous MDD variable changed to non-significant, whereas the evidence for an association between androgens and continuous depression variables (PROMIS-Depression) remained significant. If the alpha-level was lowered (and the beta-level remain) there is a need to increases the sample size [32]. This was not possible in this already completed study, thus the results of the Bonferroni-Holm indicate difficulties with i.a. low statistical power, considering that significance changed for dichotomous outcomes but not for the continuous outcome PROMIS-Depression. Nevertheless, we conclude that the only robust effect in the study was the association between DHEA and PROMIS-Depression and that longitudinal research designs in large samples are needed to understand the interplay between androgens, depression, and developmental and social factors in youth.

Additional limitations in the current study include that the study was not specifically designed to examine this particular research question focussing on puberty and the related skewed distribution of Tanner Stage and the lack of estrogen data. Additionally, there may be potential gender biases in depression diagnoses due to sex-specific symptom reporting and information on validity for the combined form of Oslo-3 and F-SozU-7 questionnaires is lacking. Lastly, considering the low number of diagnostic cases in sex-specific analyses, and the recommended minimal outcome events per predictor variable (EPV) in logistic regression models [75], results from categorical analyses has to be interpreted carefully and are less robust compared with the conducted dimensional analysis. However, the rule for EPVs in logistic regression is discussed differentially and there is evidence that fewer EPVs can be accepted when the results are interpreted carefully and in light of other factors (e.g., confidence intervals, full sample size, comparing the results with models with fewer predictors – exemplarily the age-adjusted models in this study [98]). In this regard, effect sizes for significant results in cross-sectional analyses in males were small, but still > 0.2. In longitudinal analyses, effect sizes were small or very small and had to be interpreted considering underpowered analyses.

## Conclusion

The present observational study yielded no consistent cross-sectional or longitudinal associations of hair androgen concentrations with depressive disorders in adolescents from the general population. The observed association between the cortisol/DHEA ratio and depressive symptoms in young males warrants further research. To elucidate the role of sex hormones in the pathogenesis of depressive disorders, large longitudinal studies with repeated assessments are needed to understand the complex interplay in adolescents between androgens, depression, and developmental and social factors.

## Supplementary Information

Below is the link to the electronic supplementary material.

Supplementary file1 (DOCX 42 KB)

Supplementary file2 (DOCX 40 KB)
