# Correction to: Hair androgen concentrations and depressive disorders in adolescents from the general population

**DOI:** 10.1007/s00787-022-02009-3

**Published:** 2022-05-19

**Authors:** Hanna Kische, Catharina Voss, Robin Haring, Theresa Magdalena Ollmann, Lars Pieper, Clemens Kirschbaum, Katja Beesdo-Baum

**Affiliations:** 1 Institute of Clinical Psychology and Psychotherapy, Behavioral Epidemiology, Technische Universität Dresden, Chemnitzer Str. 46, 01187 Dresden, Germany; 2 Faculty of Applied Public Health, European University of Applied Sciences, Rostock, Germany; 3 School of Public Health and Preventive Medicine, Monash University, Melbourne, Australia; 4 Center for Clinical Epidemiology and Longitudinal Studies (CELOS), Institute of Clinical Psychology and Psychotherapy, Technische Universität Dresden, Dresden, Germany; 5 Department of Biopsychology, Technische Universität Dresden, Dresden, Germany

## Correction to: European Child & Adolescent Psychiatry 10.1007/s00787-021-01929-w

The original article was published with an error in Table 1. The measuring unit for *all hair hormone data* in Table 1 should be pg/mg (see revised Table 1). The authors would like to apologise for any inconvenience caused.

**Revised table**

**Table 1:** Baseline characteristics of the study population

Variable	Total sample (*N* = 985)	Males (*N* = 412)	Females (*N* = 573)	*p*-value*
Age, years	17.9 (0.07)	18.0 (0.11)	17.9 (0.10)	0.425
Sex, female, %	48.1	0	100	
Education, %				0.190
Low	2.1	3.2	1.0	
Middle	16.8	15.8	17.7	
High	78.1	78.1	78.2	
Other	2.9	2.7	3.1	
12-Month MDD, %	4.3	3.1	5.6	0.042
12-Month MDD without any anxiety disorder, %	2.2	1.6	2.8	0.257
PHQ-9 Depression Score, mean (SD)	4.19 (0.12)	3.71 (0.17)	4.71 (0.18)	< 0.001
PROMIS Depression Score, mean (SD)	11.04 (0.16)	10.28 (0.21)	11.86 (0.23)	< 0.001
Hair testosterone, pg/mg	0.76 (0.02)	0.96 (0.04)	0.54 (0.02)	< 0.001
Change in hair testosterone, pg/mg	− 0.02 (0.04)	− 0.06 (0.06)	0.01 (0.04)	0.791
Hair DHEA, pg/mg	36.5 (1.84)	42.52 (3.29)	29.9 (1.28)	< 0.001
Change in hair DHEA, pg/mg	− 6.7 (2.47)	− 10.55 (4.58)	− 3.86 (1.8)	0.933
Hair cortisol, pg/mg	7.26 (0.47)	6.78 (0.83)	7.78 (0.39)	< 0.001
Ratio Cortisol/Testosterone	27.7 (2.92)	21.64 (5.87)	40.0 (5.28)	< 0.001
Ratio Cortisol/DHEA	0.35 (0.02)	0.29 (0.02)	0.42 (0.03)	< 0.001
Waist circumference, cm	78.1 (0.39)	80.8 (0.61)	75.1 (0.46)	< 0.001
Current smoker, %	20.6	22.4	18.6	0.253
Physically inactive, %	36.2	34.5	38.1	0.112
No alcohol use, %	20.1	18.9	21.3	< 0.001
Use of oral contraceptives, %	5.5	0	11.5	< 0.001
Oslo-3-Score, mean (SD)	10.9 (0.06)	10.7 (0.11)	11.2 (0.09)	< 0.001
F-SozU-7 Score, mean (SD)	4.19 (0.02)	4.04 (0.04)	4.35 (0.03)	< 0.001
Social Support standardized composite score, mean (SD)	− 0.012 (0.036)	− 0.227 (0.060)	0.210 (0.037)	< 0.001
Tanner Stage, %				0.056
1	–	–	–	
2	0.4	0.7	0.1	
3	4.8	5.5	4.1	
4	19.1	19.5	18.6	
5	75.6	74.1	77.1	

Data are weighted percentages, or weighted means (SE). “Any depressive disorder” includes Major Depressive Disorder and Persistent Depressive Disorder (Dysthymia). *Statistical comparisons were performed with *χ*^2^ test (nominal data) or Mann–Whitney-*U*-test (continuous data). No alcohol use: participants with response “never” on the question how often they drank alcohol in the last 3 months. The social support score is a z-standardized composite score including z-standardized Oslo-3 and z-standardized F-SozU-7

*DHEA* Dehydroepiandrosterone, *MDD* major depressive disorder, *GAD* generalized anxiety disorder, *PHQ* Patient Health Questionnaire, *PROMIS* Patient Reported Outcomes Measurement Information System, *F-SozU* Perceived Social Support Questionnaire

