# Supplementary material for: Hair androgen concentrations and depressive disorders in adolescents from the general population
Source: Eur Child Adolesc Psychiatry. 2022 Feb 2;32(8):1375–89. doi: 10.1007/s00787-021-01929-w (PMC10326161; doi:10.1007/s00787-021-01929-w)
Supplement: Supplementary file 1 — Supplementary file1 (DOCX 42 KB) [file 787_2021_1929_MOESM1_ESM.docx]

**Supplement**

**Supplementary table 1:** Characteristics of the study population (outcomes, predictors, moderators) at one-year follow-up.

| **Variable** | **Total Sample**  **(N = 515)** | **Males (N = 203)** | **Females (N = 312)** | **p-value*** |
| --- | --- | --- | --- | --- |
| **Age, years** | 19.1 (0.10) | 19.1 (0.15) | 19.0 (0.14) | 0.972 |
| **Sex, female, %** | 50.5 | 0 | 100 |  |
| **12-Month MDD** | 3.7 | 1.7 | 5.6 | 0.052 |
| **12- Month MDD without any anxiety disorder, %** | 2.5 | 1.3 | 3.7 | 0.157 |
| **PHQ-9 Score, mean (SD)** | 3.27 (0.13) | 3.02 (0.20) | 3.52 (0.16) | 0.019 |
| **Hair testosterone, ng/mg** | 0.76 (0.03) | 0.96 (0.04) | 0.56 (0.03) | <0.001 |
| **Hair DHEA, ng/mg** | 29.2 (1.21) | 33.2 (1.96) | 25.5 (1.42) | <0.001 |
| **Hair cortisol, ng/mg** | 5.41 (0.21) | 4.95 (0.33) | 5.83 (0.25) | <0.001 |
| **Ratio Cortisol/Testosterone** | 16.33 (1.70) | 10.13 (2.39) | 22.4 (2.35) | <0.001 |
| **Ratio Cortisol/DHEA** | 0.46 (0.08) | 0.29 (0.06) | 0.53 (0.14) | <0.001 |
| **Waist circumference, cm** | 78.0 (0.55) | 81.7 (0.85) | 74.3 (0.58) | <0.001 |
| **Oslo-3-Score** | 11.1 (0.13) | 11.1 (0.08) | 11.2 (0.10) | 0.169 |

Data are weighted percentages, or weighted means (SE). *Statistical comparisons were performed with χ 2 test (nominal data) or Mann-Whitney-U-test (continuous data). No alcohol use: participants with response “never” on the question how often they drank alcohol in the last three months. F-SozU was not assessed at one-year follow-up.

DHEA, Dehydroepiandrosterone. MDD, Major Depressive Disorder. GAD, Generalized Anxiety Disorder. PHQ, Patient Health Questionnaire. PROMIS, Patient Reported Outcomes Measurement Information System.

**Supplementary table 2:** Testing the interactions term sex in age-adjusted analyses.

| **Baseline** | **Testosterone** | | **DHEA** | | **Cortisol/Testosterone** | | **Cortisol/DHEA** | |
| --- | --- | --- | --- | --- | --- | --- | --- | --- |
| **MDD** | Odds Ratios (95% CI) | | | | | | | |
| **age-adjusted main effect** | 0.38 (0.13; 1.12) | | 0.96 (0.93; 0.99) | | 0.99 (0.96; 1.02) | | 0.77 (0.22; 2.64) | |
| **Interaction term sex** | 3.04 (1.09; 8.44)* | | 1.02 (1.01; 1.05)* | | 1.00 (0.97; 1.03) | | 1.41 (0.41; 4.86) | |
| **MDD without any anxiety disorder** |  | |  | |  | |  | |
| **age-adjusted main effect** | 0.35 (0.09; 1.37) | | 0.97 (0.94; 1.01) | | 0.99 (0.99; 1.02) | | 1.11 (0.57; 2.17) | |
| **Interaction term sex** | 3.30 (0.84; 12.98) | | 1.02 (0.98; 1.06) | | 1.01 (0.99; 1.07) | | 0.96 (0.45; 2.05) | |
| b-coefficients (95% CI) | | | | | | | | |
| **PHQ-9 Depression**  **Score** |  | |  |  |  |  |  |  |
| **age-adjusted main effect** | -0.001 (-0.01; 0.01) | | 0.49 (0.38; 0.60)*** | | -1.39 (-2.53; -0.26)* | | -0.009 (-0.015; -0.002)** | |
| **Interaction term sex** | 0.22 (0.12; 0.33)*** | | 0.49 (0.38; 0.60)*** | | 0.92 (0.90; 0.98)*** | | 0.88 (0.80; 0.97)*** | |
| **PROMIS Depression** |  |  |  |  |  |  |  |  |
| **age-adjusted main effect** | -0.007 (-0.01; 0.001) | | -0.36 (-1.16; 0.42) | | -0.96 (-1.76; -0.16)* | | -0.009 (-0.01; -0.005)*** | |
| **Interaction term sex** | 0.24 (0.13; 0.34)*** | | 0.50 (0.38; 0.61)*** | | 0.99 (0.91; 0.99)*** | | 0.89 (0.81; 0.97)*** | |

Data are weighted, unstandardized b-coefficients or Odds Ratios, respectively, and their 95% confidence interval, with p < 0.05 marked as *, p< 0.01 marked as **, and p<0.001 marked as ***. DHEA, Dehydroepiandrosterone. MDD, Major Depressive Disorder. GAD, Generalized Anxiety Disorder. PHQ, Patient Health Questionnaire. PROMIS, Patient Reported Outcomes Measurement Information System. CI, Confidence interval.

**Supplementary table 3:** Sex-specific cross-sectional associations of baseline hair steroid hormones with baseline depressive symptoms with **standardized Odds Ratios and β-coefficients**.

| **Baseline** | **Testosterone** | | **DHEA** | | **Cortisol/Testosterone** | | **Cortisol/DHEA** | |
| --- | --- | --- | --- | --- | --- | --- | --- | --- |
|  | **Males** | **Females** | **Males** | **Females** | **Males** | **Females** | **Males** | **Females** |
| **MDD** | Standardized Odds Ratios (95% CI) | | | | | | | |
| **age-adjusted** | 0.27  (0.05; 1.38) | 1.18  (0.86; 1.61) | 0.13  (0.009; 1.86)  ) | 0.86  (0.64; 1.15) | 0.88  (0.55; 1.41) | 0.01  (0.001; 28.21) | 1.04  (0.78; 1.38) | 0.96  (0.76; 1.20) |
| **multivariable-adjusted: Confounder** | 0.34  (0.07; 1.53) | 1.04  (0.72; 1.519 | 0.07  (0.003; 1.70) | 0.75  (0.52; 1.08) | 0.87  (0.48; 1.58) | 0.10  (0.0001; 52.69) | 1.23  (0.94; 1.60) | 0.97  (0.76; 2.15) |
| **MDD without any anxiety disorder** |  |  |  |  |  |  |  |  |
| **age-adjusted** | 0.23  (0.02; 2.02) | 1.22  (0.82; 1.61) | 0.27  (0.007; 9.89) | 1.05  (0.84; 1.33) | 0.96  (0.72; 1.28) | 0.05  (0.001; 23.21) | 1.16  (0.92; 1.47) | 0.91  (0.59; 1.42) |
| **multivariable-adjusted** | 0.25  (0.03; 1.81) | 1.25  (0.91; 1.73) | 0.25  (0.005; 12.89) | 1.05  (0.82; 1.35) | 0.92  (0.69; 1.23) | 0.13  (0.0001; 30.5) | 1.17  (0.85; 1.61) | 0.97  (0.69; 1.38) |
| Standardized β-coefficients (95% CI) | | | | | | | | |
| **PHQ-9 Depression**  **Score** |  |  |  |  |  |  |  |  |
| **age-adjusted** | 0.06  (0.01; 0.15)* | 0.02  (-0.06; 0.11) | 0.03  (-0.10; 0.16) | 0.02  (-0.04; 0.09) | 0.04  (-0.09; -0.001)* | -0.02  (-0.06; 0.01) | -0.05  (-0.13; 0.02) | -0.04  (-0.10; 0.02) |
| **multivariable-adjusted** | 0.02  (-0.06; 0.11) | 0.02  (-0.07; 0.11) | -0.03  (-0.17; 0.11) | -0.004  (-0.08; 0.07) | -0.04  (-0.08; -0.004)* | -0.03  (-0.09; 0.02) | -0.05  (-0.15; 0.04) | -0.01  (-0.08; 0.04) |
| **PROMIS Depression** |  |  |  |  |  |  |  |  |
| **age-adjusted** | 0.02  (-0.06; 0.10) | 0.01  (-0.06; 0.10) | 0.02  (-0.12; 0.17) | -0.01  (-0.08; 0.05) | -0.04  (-0.09; -0.001)* | -0.02  (-0.06; 0.01) | **-0.10**  **(-0.16; -0.03)**** | -0.01  (-0.09; 0.06) |
| **multivariable-adjusted** | 0.01  (-0.08; 0.11) | 0.005  (-0.07; 0.08) | 0.02  (-0.16; 0.21) | -0.03  (-0.10; 0.03) | -0.04  (-0.08; -0.004) | -0.03  (-0.09; 0.02) | **-0.11**  **(-0.19; -0.03)**** | -0.004  (-0.09; 0.08) |

Data are weighted standardized β-coefficients or odds rations and their 95% confidence interval, with p < 0.05 marked as *, p < 0.01 marked as **, and p < 0.001 marked as ***. The multivariable model was adjusted for Tanner Stage, waist circumference, smoking status, physical inactivity, alcohol consumption, hair color, frequency of hair cleaning, and hair treatment with heat, and oral contraceptives (females). After Bonferroni-Holm Correction only the bold marked results remain statistically significant. MDD, males N = 11, comparison group N = 401. MDD, females N = 30, comparison group N = 543. MDD, without any anxiety disorder males N = 6, comparison group N = 406. MDD, without any anxiety disorder females N = 14, comparison group N = 559. PHQ-9, males N = 412, females N = 571. PROMIS-Depression, males N = 401, females N = 565.DHEA, Dehydroepiandrosterone. MDD, Major Depressive Disorder. PHQ, Patient Health Questionnaire. PROMIS, Patient Reported Outcomes Measurement Information System. CI, confidence interval.

**Supplementary table 4:** Sex-specific longitudinal associations of baseline hair steroid hormones and change in hair steroid hormones with follow-up major depression and depressive symptoms with **standardized β-coefficients and standardized Odds Ratios**.

|  | **Testosterone** | | **DHEA** | | **Cortisol/Testosterone** | | **Cortisol/DHEA** | |
| --- | --- | --- | --- | --- | --- | --- | --- | --- |
|  | **Males** | **Females** | **Males** | **Females** | **Males** | **Females** | **Males** | **Females** |
| **FU-MDD** | **Standardized Odds Ratio (95% CI)** | | | | | | | |
| **Baseline hormone** | 1.23  (0.60; 2.53) | 1.06  (0.70; 1.58) | 0.13  (0.001; 4.52) | 0.85  (0.48; 1.50) | 0.001  (0.0001; 9.59) | 0.002  (0.001; 0.17)* | 0.80  (0.56; 1.14) | 0.38  (0.12; 1.15) |
| **Hormone change** | 0.85  (0.41; 1.74) | 0.77  (0.43; 1.36) | 0.76  (0.35 1.58) | 0.91  (0.61; 1.35) | 6.62  (0.38; 114.40) | 0.98  (0.29; 3.23) | 0.85  (0.49; 1.03) | 0.72  (0.50; 1.05) |
| **FU-MDD without any anxiety disorder** | |  |  |  |  |  |  |  |
| **Baseline hormone** | 0.85  (0.23; 3.12) | 0.88  (0.50; 1.55) | 0.03  (0.001; 3.63) | 0.76  (0.35; 1.69) | 0.001  (0.001; 0.23)* | 0.002  (0.001; 10.1) | 0.41  (0.07; 2.23) | 0.29  (0.07; 1.14) |
| **Hormone change** | 0.82  (0.45; 1.48) | 0.76*  (0.47; 1.22) | 0.82  (0.45; 1.48) | 0.77  (0.35; 1.69) | 6.85  (0.82; 56.80) | 1.77  (0.63; 5.01) | 0.76  (0.15; 2.27) | 0.39  (0.05; 2.83) |
|  | Standardized ß-coefficients (95% CI) | | | | | | | |
| **FU-PHQ-9 Depression Score** | |  |  |  |  |  |  |  |
| **Baseline hormone** | 0.02  (-0.07: 0.13) | -0.008  (-0.12; 0.11) | -0.06  (-0.25; 0.12) | 0.001  (-0.09; 0.09) | -0.04  (-0.08; -0.01) | 0.006  (-0.003; 0.01) | -0.10  (-0.25; 0.04) | -0.06  (-0.18; 0.05) |
| **Hormone change** | -0.07  (-0.22; 0.06) | -0.05  (-0.4: 0.02) | 0.04  (-0.04; 0.12) | -0.04  (-0.13; 0.04) | -0.01  (-0.13; -0.16) | 0.07  (-0.02; 0.17) | -0.42  (-0.08; 0.00) | -0.04  (-0.13; 0.03) |

Data are weighted standardized ß-coefficients or odds ratios and their 95% confidence interval, with p < 0.05 marked as *, p < 0.01 marked as **, and p < 0.001 marked as ***. Models were adjusted for Tanner Stage, waist circumference, smoking status, physical inactivity, alcohol consumption, hair color, frequency of hair cleaning, and hair treatment with heat, and oral contraceptives (females). After Bonferroni-Holm Correction only the bold marked results remain statistically significant. Hormone change is the change of hormone concentration from baseline to one-year follow-up. PROMIS-Depression was not available at FU1.

DHEA, Dehydroepiandrosterone. MDD, Major Depressive Disorder. GAD, Generalized Anxiety Disorder. PHQ, Patient Health Questionnaire. PROMIS, Patient Reported Outcomes Measurement Information System. CI, confidence interval.

| **Baseline** | **Testosterone** | | **DHEA** | |
| --- | --- | --- | --- | --- |
|  | **Males** | **Females** | **Males** | **Females** |
|  | **Odds Ratio (95% CI)** | | | |
| **MDD** |  | | | |
| First Tertile | 3.71 (0.79; 17.3) | 0.72 (0.26; 1.95) | 1.36 (0.35; 5.18) | 0.34 (0.12; 0.98)* |
| Third Tertile | 0.64 (0.09; 4.30) | 1.21 (0.26; 3.38) | 0.17 (0.35; 5.18) | 0.52 (0.18; 1.46) |
| **MDD without any anxiety** |  |  |  |  |
| First Tertile | 2.96 (0.34; 25.3) | 0.16 (0.03; 0.87)* | 2.11 (0.32; 13.8) | 0.09 (0.01; 0.75)* |
| Third Tertile | 0.50 (0.03; 6.72) | 1.08 (0.29; 3.94) | 0.58 (0.05; 6.75) | 0.99 (0.29; 3.33) |
|  | **b-coefficient (95% CI)** | | | |
| **PHQ-9** |  | | | |
| First Tertile | 0.51 (-0.55; 1.58) | 0.02 (-0.72; 0.77) | -0.34 (-1.21; 0.52) | -0.89 (-1.72; -0.06)* |
| Third Tertile | 0.33 (-0.56; 1.23) | 0.19 (-0.81; 1.19) | -0.52 (-1.39; 0.34) | -0.42 (-1.37; 0.53) |
| **PROMIS Depression** |  |  |  |  |
| First Tertile | -0.79 (-2.03; 0.44) | -0.08 (-1.27; 1.09) | -1.11 (-1.98; -0.24)* | -0.21 (-1.42; 1.01) |
| Third Tertile | -0.26 (-1.34; 0.82) | -0.08 (-1.38; 1.21) | -0.01 (-1.21; 1.11) | 0.08 (-1.18; 1.35) |
|  |  |  |  |  |

**Supplementary table 5:** Sex-specific cross-sectional associations of baseline hair androgen terciles with baseline depression and depressive symptoms.

Data are weighted, unstandardized b-coefficients or Odds Ratios, respectively, and their 95% confidence interval, with p < 0.05 marked as *, p< 0.01 marked as **, and p<0.001 marked as ***. After Bonferroni-Holm Correction none of the results remained statistically significant. The second tertile was used as reference. Models were adjusted for Tanner Stage, waist circumference, smoking status, physical inactivity, alcohol consumption, hair color, frequency of hair cleaning, and hair treatment with heat. DHEA, Dehydroepiandrosterone. MDD, Major Depressive Disorder. GAD, Generalized Anxiety Disorder. PHQ, Patient Health Questionnaire. PROMIS, Patient Reported Outcomes Measurement Information System. CI, Confidence interval.

**Supplementary table 6:** Interaction of social support in the sex-specific association of baseline depression and baseline androgens.

| **Baseline** | **Testosterone** | | **DHEA** | | **Cortisol/Testosterone** | | **Cortisol/DHEA** | |
| --- | --- | --- | --- | --- | --- | --- | --- | --- |
|  | **Males** | **Females** | **Males** | **Females** | **Males** | **Females** | **Males** | **Females** |
| **MDD** | **Odds Ratio (95% CI)** | | | | | | | |
| Main effect | 0.32  (0.06; 1.61) | 1.33  (0.61; 2.91) | 0.96  (0.92; 1.00) | 0.99  (0.98; 1.01) | 0.98  (0.93; 1.04) | 0.99  (0.98; 1.01) | 1.47  (0.52; 4.11) | 0.93  (0.41; 2.14) |
| Interaction with social support | 2.20  (0.99; 4.83) | 0.79  (0.38; 1.64) | 1.02  (1.01; 1.04)* | 0.99  (0.97; 1.02) | 0.98  (0.94; 1.03) | 0.99  (0.98; 1.01) | 0.37  (0.08; 1.64) | 0.71  (0.27; 1.88) |
| **MDD without any anxiety disorder** | | | | | | | | |
| Main effect | 0.21  (0.01; 2.65) | 1.73  (0.79; 3.78) | 0.95  (0.89; 1.03) | 1.01  (0.99; 1.02) | 0.99  (0.98; 1.01) | 0.99  (0.98; 1.01) | 1.30  (0.70; 2.43 | 0.79  (0.57; 1.66) |
| Interaction with social support | 1.24  (0.36; 4.20) | 0.63  (0.29; 1.39) | 1.03  (0.98; 1.09) | 0.99  (0.97; 1.01) | 0.99  (0.98; 1.01) | 0.98  (0.97; 1.01) | 0.99  (0.99; 1.02) | 0.99  (0.98; 1.01) |
| **PHQ-9** | **b-coefficients (95% CI)** | | | | | | | |
| Main effect | 0.31  (-0.10; 0.63) | 0.44  (-0.41; 1.31) | 0.001  (-0.006; 0.008) | 0.01  (-0.001; 0.02) | -0.001  (-0.0012; -0.0004)* | -0.0001  (-0.0001; -0.00001)* | 0.19  (-0.69; 1.08) | -0.17  (-0.73; 0.37) |
| Interaction with social support | 0.33  (-0.18; 0.85) | -0.88  (-1.77; 0.01) | -0.0001  (-0.006; 0.006) | -0.011  (-0.02; 0.003) | 0.0006  (-0.0003; 0.001) | -0.0001  (-0.0003; 0.0001) | -0.64  (-1.97; 0.67) | 0.05  (-0.58; 0.69) |
| **PROMIS Depression** | |  |  |  |  |  |  |  |
| Main effect | 0.31  (-0.18; 0.82) | 0.52  (-0.63; 1.69) | 0.15  (0.007; 0.29)* | 0.14  (-0.05; 0.33) | -0.001  (-0.001; -0.0002)* | -0.00005  (-0.00001;0.0001) | -0.84  (-1.67; -0.01)* | 0.51  (-0.64; 1.68) |
| Interaction with social support | 0.62  (-0.18; 1.43) | -0.91  (-2.27; 0.43) | -0.005  (-0.02; 0.01) | -0.003  (-0.02; 0.01) | 0.0007  (-0.0004; 0.001) | 0.00005  (-0.0001; 0.0002) | 0.91  (-0.48; 2.31) | -0.91  (-2.29; 0.47) |

Data are weighted, unstandardized b-coefficients or Odds Ratios, and their 95% confidence interval, with p < 0.05 marked as *, p< 0.01 marked as **, and p<0.001 marked as ***. After Bonferroni-Holm Correction none of the results remained statistically significant. Models were adjusted for Tanner Stage, waist circumference, smoking status, physical inactivity, alcohol consumption, hair color, frequency of hair cleaning, and hair treatment with heat. Results are additive interactions (change of risk difference between androgens and depression if the composite scale for social support increases by 1). The social support score is a z-standardized composite score including z-standardized Oslo-3 and z-standardized F-SozU-7. DHEA, Dehydroepiandrosterone. MDD, Major Depressive Disorder. GAD, Generalized Anxiety Disorder. PHQ, Patient Health Questionnaire. PROMIS, Patient Reported Outcomes Measurement Information System. CI, confidence interval**.**
