# Supplementary material for: Hair androgen concentrations and depressive disorders in adolescents from the general population
Source: Eur Child Adolesc Psychiatry. 2022 Feb 2;32(8):1375–89. doi: 10.1007/s00787-021-01929-w (PMC10326161; doi:10.1007/s00787-021-01929-w)
Supplement: Supplementary file 2 — Supplementary file2 (DOCX 40 KB) [file 787_2021_1929_MOESM2_ESM.docx]

**Supplement 2:**

**Additional information on sensitivity analyses**

**Limitations:**

There is no strong evidence that oral inhalative corticoid medication result in systemic changes of steroid hormone concentrations (Morrison et al., 1994). Thus, these participants were not excluded from main analyses. However, it might be that this medication use refers to diseases that might be associated with alterations in steroid hormone concentrations, but this couldn’t be proofed with our study data. To ensure no effect is overlooked, we adjusted one model in sensitivity analyses for inhalative corticoid medication.

In regard of pregnancy, there was no question available referring to current pregnancy. We used the self-reported answer to the question “Are you pregnant or have you ever been pregnant?”. Thus, if the answer to this question was “yes” it could be that the particular participant was still currently not pregnant. We decided that it would not be appropriate to exclude these participants based on this question in the main analyses. However, to ensure no effect is overlooked, we adjusted one model in sensitivity analyses for possible pregnancy.

**References for the used confounders:**

We used variables with high evidence from previous literature for being confounders in the main analyses (Granger et al., 2003) (age (Ilondo et al., 1982, Lewinsohn et al., 1994) or Tanner Stage (Angold et al., 1999, Angold et al., 1998), respectively, waist circumference (Soares et al., 2019, Svartberg et al., 2004), smoking status (Chaiton et al., 2009, Kische et al., 2016), alcohol consumption (Apter and Eriksson, 2003, Galaif et al., 2007), physical inactivity (Bang et al., 2020, Rothon et al., 2010), hair-associated confounders (due to cortisol measured in hair (Stalder and Kirschbaum, 2012)), and oral contraceptives (only in analyses with females (de Wit et al., 2020, Søeborg et al., 2014))). Second, we added four specific confounders to the multivariable models, respectively: possible pregnancy (Bammann et al., 1980, Siegel and Brandon, 2014), menstruation status (Dorn et al., 2009, Lobotsky et al., 1964), use of inhalated corticoid medication (Morrison et al., 1994, Opolski and Wilson, 2005) and use of antidepressants (Giltay et al., 2012, Jureidini et al., 2004). These confounders have been found to have an impact on the outcomes and predictors (= confounder), but either with no high evidence (for one of them) and not with such high effects compared to the other confounders initially used in the main multivariable analyses or the variables itself had limitations (Supplement 2). Therefore, these confounders were only included in sensitivity analyses.

**References:**

Angold A, Costello EJ, Erkanli A, Worthman CM. Pubertal changes in hormone levels and depression in girls. Psychological medicine 1999;29(5):1043-53.

Angold A, Costello EJ, Worthman CM. Puberty and depression: the roles of age, pubertal status and pubertal timing. Psychological medicine 1998;28(1):51-61.

Apter SJ, Eriksson CJ. The effect of alcohol on testosterone concentrations in alcohol-preferring and non-preferring rat lines. Alcohol Clin Exp Res 2003;27(7):1190-3.

Bammann BL, Coulam CB, Jiang NS. Total and free testosterone during pregnancy. Am J Obstet Gynecol 1980;137(3):293-8.

Bang H, DoyeonWon, Park S. School engagement, self-esteem, and depression of adolescents: The role of sport participation and volunteering activity and gender differences. Children and Youth Services Review 2020.

Chaiton MO, Cohen JE, O'Loughlin J, Rehm J. A systematic review of longitudinal studies on the association between depression and smoking in adolescents. BMC public health 2009;9:356.

de Wit AE, Booij SH, Giltay EJ, Joffe H, Schoevers RA, Oldehinkel AJ. Association of Use of Oral Contraceptives With Depressive Symptoms Among Adolescents and Young Women. JAMA psychiatry 2020;77(1):52-9.

Dorn LD, Negriff S, Huang B, Pabst S, Hillman J, Braverman P, et al. Menstrual symptoms in adolescent girls: association with smoking, depressive symptoms, and anxiety. J Adolesc Health 2009;44(3):237-43.

Galaif ER, Sussman S, Newcomb MD, Locke TF. Suicidality, depression, and alcohol use among adolescents: a review of empirical findings. International journal of adolescent medicine and health 2007;19(1):27-35.

Giltay EJ, Enter D, Zitman FG, Penninx BW, van Pelt J, Spinhoven P, et al. Salivary testosterone: associations with depression, anxiety disorders, and antidepressant use in a large cohort study. Journal of psychosomatic research 2012;72(3):205-13.

Granger DA, Shirtcliff EA, Zahn-Waxler C, Usher B, Klimes-Dougan B, Hastings P. Salivary testosterone diurnal variation and psychopathology in adolescent males and females: individual differences and developmental effects. Development and psychopathology 2003;15(2):431-49.

Ilondo MM, Vanderschueren-Lodeweyckx M, Vlietinck R, Pizarro M, Malvaux P, Eggermont E, et al. Plasma androgens in children and adolescents. Part I: control subjects. Horm Res 1982;16(2):61-77.

Jureidini JN, Doecke CJ, Mansfield PR, Haby MM, Menkes DB, Tonkin AL. Efficacy and safety of antidepressants for children and adolescents. Bmj 2004;328(7444):879-83.

Kische H, Gross S, Wallaschofski H, Völzke H, Dörr M, Nauck M, et al. Clinical correlates of sex hormones in women: The study of health in Pomerania. Metabolism: clinical and experimental 2016;65(9):1286-96.

Lewinsohn PM, Clarke GN, Seeley JR, Rohde P. Major depression in community adolescents: age at onset, episode duration, and time to recurrence. Journal of the American Academy of Child and Adolescent Psychiatry 1994;33(6):809-18.

Lobotsky J, Wyss HI, Segre EJ, Lloyd CW. PLASMA TESTOSTERONE IN THE NORMAL WOMAN. The Journal of clinical endocrinology and metabolism 1964;24:1261-5.

Morrison D, Capewell S, Reynolds SP, Thomas J, Ali NJ, Read GF, et al. Testosterone levels during systemic and inhaled corticosteroid therapy. Respiratory medicine 1994;88(9):659-63.

Opolski M, Wilson I. Asthma and depression: a pragmatic review of the literature and recommendations for future research. Clin Pract Epidemiol Ment Health 2005;1:18.

Rothon C, Edwards P, Bhui K, Viner RM, Taylor S, Stansfeld SA. Physical activity and depressive symptoms in adolescents: a prospective study. BMC Med 2010;8:32.

Siegel RS, Brandon AR. Adolescents, pregnancy, and mental health. J Pediatr Adolesc Gynecol 2014;27(3):138-50.

Soares FC, Barros MVG, Bezerra J, Santos SJ, Machado L, Lima RA. The synergic relationship of social anxiety, depressive symptoms and waist circumference in adolescents: Mediation analysis. Journal of affective disorders 2019;245:241-5.

Søeborg T, Frederiksen H, Mouritsen A, Johannsen TH, Main KM, Jørgensen N, et al. Sex, age, pubertal development and use of oral contraceptives in relation to serum concentrations of DHEA, DHEAS, 17α-hydroxyprogesterone, Δ4-androstenedione, testosterone and their ratios in children, adolescents and young adults. Clin Chim Acta 2014;437:6-13.

Stalder T, Kirschbaum C. Analysis of cortisol in hair--state of the art and future directions. Brain, behavior, and immunity 2012;26(7):1019-29.

Svartberg J, von Mühlen D, Sundsfjord J, Jorde R. Waist circumference and testosterone levels in community dwelling men. The Tromsø study. European journal of epidemiology 2004;19(7):657-63.
